# A Phosphorous-Based Bi-Functional Flame Retardant Based on Phosphaphenanthrene and Aluminum Hypophosphite for an Epoxy Thermoset

**DOI:** 10.3390/ijms231911256

**Published:** 2022-09-24

**Authors:** Bo Xu, Yanting Liu, Simiao Wei, Siheng Zhao, Lijun Qian, Yajun Chen, Hao Shan, Qinglei Zhang

**Affiliations:** 1 School of Chemistry and Materials Engineering, Beijing Technology and Business University, No. 11, Fucheng Road, Haidian District, Beijing 100048, China; 2 Beijing Key Laboratory of Quality Evaluation Technology for Hygiene and Safety of Plastics, No. 11, Fucheng Road, Haidian District, Beijing 100048, China; 3 China Light Industry Engineering Technology Research Center of Advanced Flame Retardants, No. 11, Fucheng Road, Haidian District, Beijing 100048, China; 4 Petroleum and Chemical Industry Engineering Laboratory of Non-halogen Flame Retardants for Polymers, No. 11, Fucheng Road, Haidian District, Beijing 100048, China; 5 China Metallurgical Information and Standardization Research Institute, No. 11, Deng Shi Kou Street, Dongcheng District, Beijing 100730, China

**Keywords:** flame retardant, intramolecular P-P synergy, aluminum phosphinate, phosphophenanthrene, epoxy resin

## Abstract

A phosphorous-based bi-functional compound HPDAl was used as a reactive-type flame retardant (FR) in an epoxy thermoset (EP) aiming to improve the flame retardant efficiency of phosphorus-based compounds. HPDAl, consisting of two different P-groups of aluminum phosphinate (AHP) and phosphophenanthrene (DOPO) with different phosphorous chemical environments and thus exerting different FR actions, exhibited an intramolecular P-P groups synergy and possessed superior flame-retardant efficiency compared with DOPO or AHP alone or the physical combination of DOPO/AHP in EP. Adding 2 wt.% HPDAl made EP composites acquire a LOI value of 32.3%, pass a UL94 V-0 rating with a blowing-out effect, and exhibit a decrease in the heat/smoke release. The flame retardant modes of action of HPDAl were confirmed by the experiments of the scanning electron microscope (SEM), X-ray photoelectron spectroscopy (XPS), and thermogravimetry–Fourier transform infrared spectroscopy–gas chromatograph/mass spectrometer (TG-FTIR-GC/MS). The results indicate that the phosphorous-based FRs show different influences on the flame retardancy of composites, mainly depending on their chemical structures. HPDAl had a flame inhibition effect in the gas phase and a charring effect in the condensed phase, with a well-balanced distribution of P content in the gas/condensed phase. Furthermore, the addition of HPDAl hardly impaired the mechanical properties of the matrix due to the link by chemical bonds between them.

## 1. Introduction

Phosphorus-based compounds, as promising halogen-free flame retardant candidates, have increasingly attracted extensive attention [1,2,3,4,5,6]. Generally speaking, previous results have shown that phosphorus flame retardant can work in both the condensed and gas phase, whilst its relative predominance depends on the chemical environment of the P atom (phosphorus valency or oxidation state, and the nature of chemical moieties surrounding the phosphorus atom) and the structural characteristics of the matrix [7,8]. The increasing oxygen environment of the P atom and its higher phosphorus valency in compounds result in an increasing charring effect, which is caused by the fact that the produced polyphosphoric acid catalyzes the formation of a protective carbonaceous layer, i.e., a condensed-phase flame retardant mechanism. On the other hand, an increasing carbon or hydrogen environment of the P atom and lower phosphorus valency contribute to a flame inhibition effect through a radical trapping mechanism, which is a predominantly gas-phase flame retardant activity, as previously mentioned in many studies [6,9]. These flame retardants decompose to produce small low-energy phosphorus-based radicals quenching the high-energy hydroxyl or hydrogen radicals from polymer decomposition in the vapor phase, thus interrupting the flame propagation. Certainly, the ability of a phosphorus-based flame retardant to be in the vapor phase or condensed phase is also a function of what type of polymer it is put into, in addition to its chemical structure. Previously, our lab constructed a series of novel bi-group molecules, which exhibited a remarkable intramolecular group synergistic effect when the groups were integrated into one molecule [10,11,12]. Investigations of this synergy would provide insights into how the flame retardant efficiency of phosphorus-based compounds could be improved. Indeed, some works have reported that one flame retardant molecule consisting of two different phosphorus-based groups with different phosphorus valency could obtain a group or P-P synergy of modes of action [13]. Phosphophenanthrene (DOPO) and phosphonitrile groups were introduced into the same molecule in the form of chemical bonding, named HAP-DOPO, for flame retardant epoxy resin, which can form phosphorus-rich residues carbon while releasing PO·, showing a better flame retardant effect than when added alone or physical mixed [14]. A bi-functional compound containing two phosphorus-based moieties—DOPO and phosphonate—was prepared, and the two phosphorus-containing groups decomposed in sequence with increasing temperature, continuously releasing PO free radicals with a quenching effect in the gas phase and forming viscous residues to bond and integrate other char species with a high barrier effect in the condensed phase, enhancing the flame retardancy of matrix [15]. However, the amount of added flame retardants was sufficiently high to achieve the desired flame-retardant properties, i.e., low flame retardant efficiency.

It is known that the development of highly effective flame retardant systems requires the design of molecules that, with low addition, have the ability to promote charring in the solid phase and simultaneously exert an effective gas-phase disruption of flame propagating reactions [16]. The balanced bi-phase modes of action may be more important for elevating the flame retardant efficiency and finally decreasing the loading amounts of FRs. A synergistic effect has been demonstrated between DOPO and the alkyl aluminum hypophosphite added to epoxy resins in the form of a complex [17]. Hence, in this study, a reactive phosphorous-based bi-functional flame retardant HPDAl was designed and prepared by integrating hypophosphite and phosphaphenanthrene (DOPO) moieties into one molecule. The two are known to have different chemical environments for the P atom and then different flame retardant mechanisms [18,19]. The intramolecular joint action of the two different P-groups was investigated in the FR efficiency for EP. Simultaneously, to better clarify the P-P synergistic FR behavior of HPDAl, comparison studies on the flame retardancy of EP samples containing DOPO or AHP separately or physical combined were carried out in this work.

## 2. Results and Discussion

### 2.1. Characterization of HPDAl

**Figure 1:**
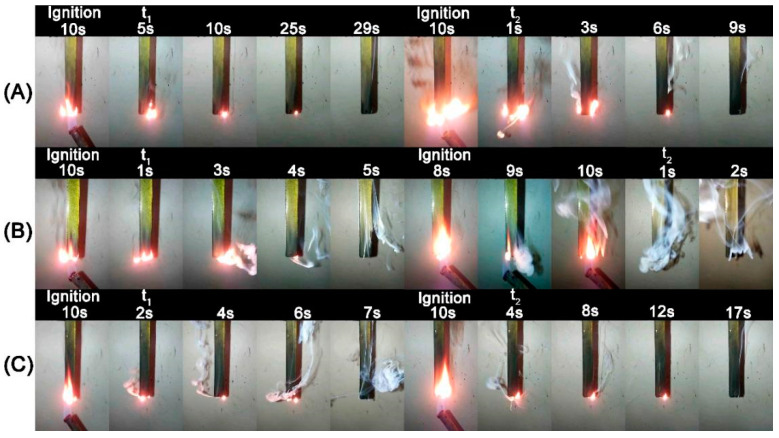
Video screenshots of a vertical burning test of 1%HPDAl/EP (**A**); 2%HPDAl/EP (**B**); and 3%HPDAl/EP (**C**).

The FTIR spectrum of HPDAl was presented in Figure 1c. Bands at 2924 and 2854 cm^−1^ were the stretching vibration of C-H on the methylene group. The peaks at 1447 to 1632 cm^−1^ were associated with the aromatic skeleton. The peak at 2387 cm^−1^ illustrated the presence of the terminal group P-H. The absorption bands at 1431 and 1200 cm^−1^ correspond to P-C and P=O, respectively. It should be noticed that the absorption band for aldehyde at 1699 cm^−1^ disappeared, while a new peak at 3423 cm^−1^ pointed to -OH arose, which initially confirmed the chemical structure of HPDAl.

**Figure 2:**
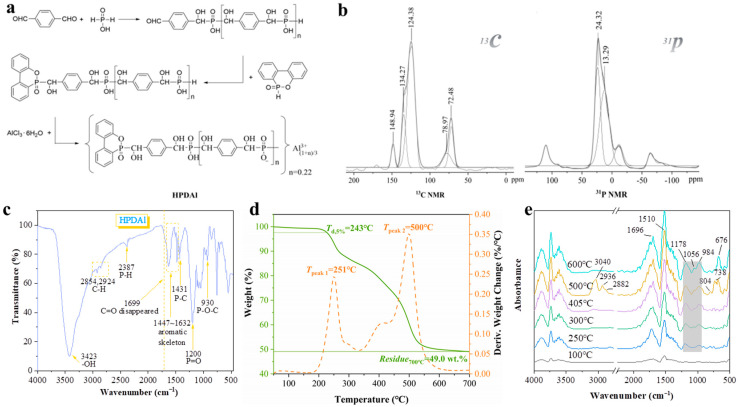
Synthesis route of HPDAI (**a**); ^13^C NMR and ^31^P NMR spectra of HPDAI (**b**); FTIR spectrum of HPDAI (**c**); TGA and DTG curves of HPDAI in N_2_ atmosphere (**d**); and TG-FTIR curves of HPDAl in N_2_ atmosphere (**e**).

In order to further clarify the chemical structure of HPDAl, ^13^C SSNMR and ^31^P SSNMR were used to examine the atoms in different chemical environments. As shown in Figure 2b, the fitting curves of the ^13^C SSNMR spectra exhibited an absorption peak at 148.94 ppm, which corresponded to the C-O of DOPO, and the peaks at 134.27 and 124.38 ppm were ascribed to the C=C bond of the benzene ring. The bands at 78.97 and 72.48 ppm were caused by P_DOPO_-CH-benzene and P_AHP_-CH-benzene, respectively. As for the ^31^P SSNMR spectrum, the peaks at 24.32 and 13.29 ppm were corresponded to C-P-C and C-P-H, respectively. In general, all of these SSNMR data indicated that HPDAl had been synthesized successfully.

Additionally, the thermal decomposition process of HPDAl was investigated by TG-FTIR. From Figure 2d, the initial decomposition temperature of HPDAl was 243 °C, and the residual char yield at 700 °C reached 49.0 wt.%, which indicated that the flame retardant itself had good thermal stability and char forming ability. Evidently, the thermal decomposition of HPDAl underwent two processes. In order to understand the role of two-stage pyrolysis products in the gas phase, the real-time gas-phase infrared testing of HPDAl was performed and the structure information of pyrolysis gases at different temperatures was shown in Figure 2e. This revealed that phosphorus oxygen P-O and P=O groups (1178 and 1056 cm^−1^) began to appear at approximately 250 °C [20], with an absorption peak intensity of the aromatic ring (at approximately 1510 cm^−1^) gradually increasing until 600 °C. The characteristic peaks of the aldehyde group (at approximately 1696 cm^−1^) were easily observed, caused by the thermal degradation of CH-OH. At 500 °C, other pyrolysis products, such as olefins (at 3040, 2936 and 2882 cm^−1^), benzene (at 676 cm^−1^), and substituted benzenes (at 738 and 804 cm^−1^), were detected. The results indicate that HPDAl was able to continuously release phosphorus–oxygen fragments at a lower temperature (approximately 250 °C) and they kept a high concentration with increasing temperature [21]. It was presumed to be the result of the volatile phosphorus–oxygen components generated by HPDAl during the combustion process being wrapped by the char layer which ruptured over time, and the aggregated volatile phosphorus–oxygen fragments were released together with other pyrolysis gases under the char layer, consequently leading to the continuous release of free radicals throughout the combustion process, which continuously exerted the free radical quenching effects [22].

### 2.2. Flammability Analysis

Vertical burning (UL 94) and LOI tests were employed to assess the flammability of flame-retardant epoxy composites. It can be seen from Table 1 that 2%HPDAl/EP obtained a value of 32.3%, higher than the other FR systems containing the physical combination of DOPO and AHP with the same P content in composites, which showed an intramolecular P-P synergy between DOPO and AHP moieties connected by chemical bonds in HPDAl.

**Table 1:** Flammability of flame-retardant epoxy composites.

Samples	LOI (%) ± 1.0	UL 94			
		Dripping	Ranking	av-t_1_ (s)	av-t_2_ (s)
EP	26.2	Yes	NR	145.6	infinite
1%HPDAl/EP	30.4	No	NR	23.1	7.4
2%HPDAl/EP	32.3	No	V-0	3.1	2.9
3%HPDAl/EP	32.3	No	V-1	10.0	22.3
AHP/EP	28.8	No	NR	95.8	18.0
DOPO/EP	30.3	No	NR	22.6	35.7
AHP/DOPO/EP	29.8	No	NR	16.2	52.2

Meanwhile, just 2%HPDAl/EP reached the UL 94 V-0 rating among all the formulations, further illustrating that the intramolecular P-P synergy more significantly lifted the flame retardant efficiency of HPDAl in EP. To elaborate this result, videos of the samples during the vertical burning test were taken and the video screenshots were shown in Figure 1. As reported by researchers, the surface flame can be blown out during combustion, which was the so-called blowing-out effect [23,24,25]. Apparently, there were some differences in the combustion phenomenon of EP composites with various HPDAl contents. The flame of 1%HPDAl/EP gradually shrank after being ignited for the first time, but the flame duration was long and there was no blowing-out effect. This may be because, from the low flame-retardant content, the char layer formed during the first ignition was not strong enough to accumulate the pyrolysis volatiles and the released phosphorus–oxygen free radicals to extinguish the flame [26]. This was likewise illustrated by the blowing-out effect from the accumulated pyrolysis volatiles containing the phosphorus–oxygen free radical due to the increased char residues during the second ignition. In contrast, 3%HPDAl/EP had a blowing-out effect at the first ignition time, but for the second ignition time, the flame did not extinguish quickly and the flame lasted for a long time. This was because the increase in flame retardant content locked more phosphorus-containing components in residues, as proven by the following XPS results, and formed a P-rich char layer mixed with aluminum pyrophosphate [27,28]. After the second ignition, it was too tough for the generated pyrolysis products to break through the excessively strong carbon layer, so that the quenching effect of phosphorus-containing free radicals cannot be effectively exerted, resulting in a weakened blowing-out effect. Then, it is not difficult to explain why 2%HPDAl/EP displayed a notable blowing-out effect after both the first and second ignition, and the flame was quickly extinguished, achieving a UL 94 V-0 rating. This resulted from the formation of a moderately strong carbon layer that not only prevented the diffusion of heat under the substrate, but can also be breached when a certain amount of pyrolysis volatiles have accumulated, thereby extinguishing the flame [29]. The blowing-out effect exerted by 2 wt% HPDAl in EP predominantly depended on an appropriate match between the formation rate/amount of pyrolysis products in gas phase and the char viscosity/strength in condensed phase [30].

### 2.3. Thermal Decomposition Behavior Analysis

**Figure 3:**
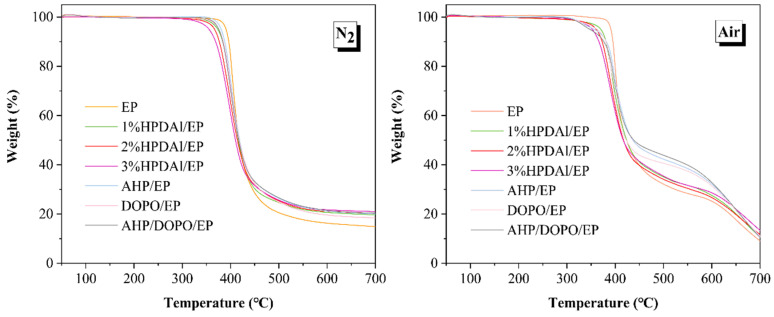
TGA curves of EP composites.

The thermal decomposition behavior of the pure EP and EP composites was investigated and the results are shown in Figure 3 and Table 2. It was revealed that the addition of all flame retardants lowered the initial decomposition temperature of EP and increased the char residues to varying degrees. Moreover, with an increasing HPDAl content, the initial decomposition temperature of composites tended to decrease and the residual carbon yield at high temperature increased accordingly. Even 3 wt.% loading of HPDAl caused a significant increase in the composite residual char yield, approximately 40% higher than that of pure EP. These results account for why adding different amounts of HPDAl resulted in different blowing-out effects, as shown by the UL 94 tests. In addition, compared with DOPO/EP and AHP/EP, 2%HPDAl/EP and AHP/DOPO/EP possessed more carbon residues and further investigation showed a synergistic charring effect, promoting the matrix to form more high-temperature thermal-stable char residues regardless of the combination type, intramolecular or intermolecular, between DOPO and AHP groups. It was also worth mentioning that the thermal decomposition behaviors of pure EP and EP composites under nitrogen and air atmospheres were approximately the same, but a new degradation stage appeared at 600 °C under air, which can be assigned to the further decomposition of the char residue.

### 2.4. Fire Behaviour: Forced Combustion

As a means of comprehensively monitoring the forced combustion process of materials under specific external heat radiation, the cone calorimeter test provides plenty of necessary real-time parameters for heat release, smoke production and toxic emissions throughout the combustion process. Some significant data are listed in Table 3.

The changing trend in the time to ignition (TTI) indicates that there was almost no effect on TTI for AHP/EP compared to EP, but TTI was significantly shorter for DOPO/EP. Meanwhile, the TTI of HPDAl/EP was earlier than that of EP and decreased further with the increasing addition of HPDAl into the composites. The reason for this phenomenon is tentatively speculated to be due to the early decomposition of DOPO in HPDAl, inducing the early decomposition of the matrix [13], while the early release of fuels led to the decrease in TTI, which can be laterally verified with the TTI data of DOPO/EP.

**Figure 4:**
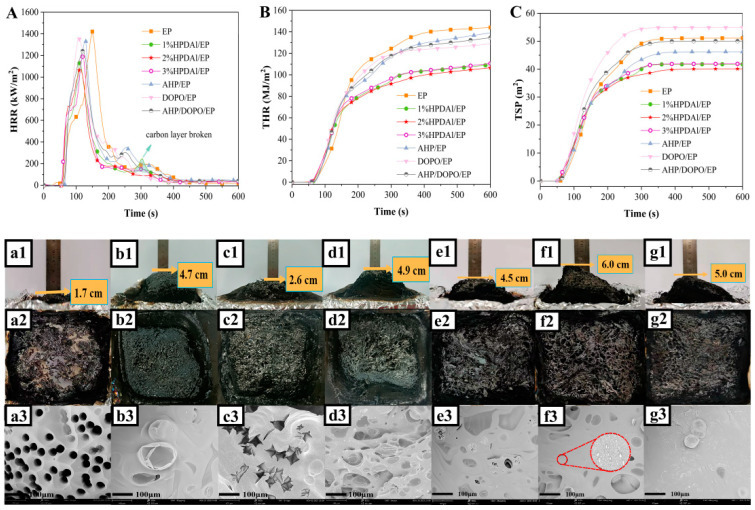
HRR (**A**), THR (**B**) and TSP (**C**) curves of EP and EP composites, macroscopic photos and SEM images (×500) of residues for EP (**a1**–**a3**), AHP/EP (**b1**–**b3**), DOPO/EP (**c1**–**c3**), AHP/DOPO/EP (**d1**–**d3**), 1%HPDAl/EP (**e1**–**e3**), 2%HPDAl/EP (**f1**–**f3**) and 3%HPDAl/EP (**g1**–**g3**) after cone test.

As shown in Figure 4 and Table 3, the peak heat release rate (PHRR) of the composite after adding DOPO or AHP separately slightly decreased compared to pure EP. After physically mixing DOPO and AHP, the PHRR value of composite was lower than that of EP/DOPO or EP/AHP with the same P content, indicating a synergistic effect between DOPO and AHP in suppressing the combustion intensity of the matrix. This phenomenon was further improved with the introduction of HPDAl. The PHRR and total heat release (THR) values of EP composites were reduced compared with those of EP/DOPO/AHP. As seen from Figure 4, the HRR curves of EP/HPDAl rapidly reached its highest value in the first rising period and the values were higher than that of pure EP. Similarly, the THR values of HPDAl/EP were higher than that of pure EP at the beginning of the combustion, but the values no longer changed as the combustion reaction proceeded to a certain extent. This indicated that the heat release of HPDAl/EP systems was more violent than that of pure EP in the early stage of combustion, which is due to the early decomposition of composites reflected by TGA results. This early degradation generated volatile pyrolysis fragments and the phosphorus–oxygen-free radicals from DOPO moieties pyrolysis in HPDAl were simultaneously released together with the pyrolysis gases, which facilitated the gas-phase quenching effect. It was reflected from the decrease in the average effective heat of combustion (av-EHC) values at the same time. With the development of the thermal decomposition process and the rising decomposition temperature, AHP started to decompose and promoted matrix char formation, whilst the synergistic effect with DOPO decreased the PHRR and THR of EP composites by 20.6%–24.5% and 23%–25% compared to pure EP, respectively. Of course, this high-quality carbon layer further prevented more residual carbon flakes from volatilizing into the air, thus suppressing the total smoke production (TSP) and total smoke release (TSR). Consequently, an intramolecular synergy was exhibited through combining DOPO and AHP groups in the form of chemical bonds in one compound and then exerted a positive effect on the flame-retardant behavior of EP composites, which cannot be effectively achieved by the physical mixing of AHP and DOPO.

For DOPO, its free radical quenching effect always leads to an increase in the incomplete combustion of matrix materials, thus forming non-combustible smoke in the gas phase and then resulting in some unavoidable elevation in TSP and TSR [31]. Additionally, this incomplete combustion was responsible for the increase in the average CO yield (av-COY) and the decrease in the average CO_2_ yield (av-CO_2_Y), which fundamentally weakened the combustion behavior. In contrast, AHP predominantly exerted the flame retardant action in condensed phase which then decomposed at high temperature to produce aluminum polyphosphate and pyrophosphate, etc. These exerted a dehydration function during the combustion process, thus promoting the matrix char formation and lessening the smoke spillage [27]. When HPDAl was added at 1 wt.%, a small peak in HRR curve was evident at approximately 300 °C. This may be due to the fact that the carbon layer was not strong enough and thus easily broken through by the internal gases. However, the carbon layer of 3%HPDAl/EP was so strong that it tightly wrapped the internal pyrolysis products, not conducive to perform the blowing-out effect and free radical quenching effect. When 2 wt.% HPDAl was added, it generated carbon residues with the moderate strength without disturbing the gas-phase flame-retardant action, i.e., an optimal balance in exerting the flame retardant effect between the gas phase and condensed phase, so that the better flame retardant properties were obtained than those of the two others.

To more distinctly explain the action route of the flame retardant effect, the quantitative assessment for three main flame-retardant modes of action, i.e., flame-inhibition effect, charring effect, barrier and protective effect, of the FRs by analyzing the results from cone calorimeter was carried out. The calculating method was shown in Equations (1)–(3) and the results are presented in Table 4 [32].

(1) $$Flame\;inhibition\;effect=(1-\frac{EHC_{fr}}{EHC_{pure}})\times 100\%$$

(2) $$Charring\;effect=(1-\frac{TML_{fr}}{TML_{pure}})\times 100\%$$

(3) $$Barrier\;and\;protective\;effect=\;(1-\frac{\frac{PHRR_{fr}}{PHRR_{pure}}}{\frac{THR_{fr}}{THR_{pure}}})\times 100\%$$

2%HPDAl/EP possessed the best flame inhibition effect compared to other FR systems containing the physically mixed system AHP/DOPO/EP, although both had the same phosphorus content. These results confirm the intramolecular synergistic effect between AHP/DOPO in HPDAl in the gaseous phase. The charring effect of 2%HPDAl/EP (7.4%) was better than 4.7% of DOPO/EP but a little worse than 8.3% of AHP/EP. Although the charring effect of 2%HPDAl/EP was located between DOPO/EP and AHP/EP, the results still show a synergistic effect if we calculate the synergistic effect according to the corresponding ratio of the AHP and DOPO moieties in HPDAl. Moreover, it was noteworthy that the charring effect of 2%HPDAl/EP was higher than the physical combination system, including the flame-inhibition effect. This indicates that the intramolecular P-P synergy generated by the chemical bonding in one compound was more effective than the intermolecular one, i.e., the physically mixed system. Furthermore, all the samples exhibited a weak barrier and protective effect with low calculated values or negative values. In sum, the flame inhibition effect and charring effect of HPDAl through the intramolecular P-P synergy principally exerted their actions to increase the flame retardancy of EP composites.

### 2.5. Morphologies of Final Char

Figure 4 displays digital photos of the EP and EP composites’ char residues obtained from cone calorimeter test. After forced combustion, the pure EP left a thin layer of broken chars, revealing its seriously poor char-forming ability. The carbon residue yields and swelling ratios increased after adding AHP or DOPO separately or physically mixed. Furthermore, the HPDAl/EP composites retained more char residues, and their surface exhibited a hard structure like a metal mesh. Many residues were superimposed inside the char layer, showing a swollen morphology. It is worth noting that the residues of 2%HPDAl/EP exhibited the largest expansion, but the expansion volume decreased with increasing amount of added HPDAl to 3 wt.%. This may be due to the characteristics of phosphorus-containing flame retardants, which endowed the residual char with a certain viscosity during combustion and this viscosity often increased with the increase in P content [33]. The appropriate viscosity/strength for residues was necessary to efficiently exert the blown-out effect. Maybe the viscosity of 3%HPDAl/EP residues was excessively high so that the internal pyrolysis products could not break through the formed carbon layer and escape to the outside in time, while 1%HPDAl/EP residues was excessively low to accumulate the internal pyrolysis gases. Gratifyingly, 2%HPDAl/EP exhibited just the right balance of the gas release and char quality to fully exert the blowing-out effect. In other words, the char layer had a certain strength, allowing phosphorus–oxygen free radicals to rush out along with the pyrolysis products, and the gas-phase and condensed-phase mechanisms cooperated with each other, subsequently gaining a better flame-retardant effect.

In addition, the microscopic morphology of EP and EP composites residues observed by SEM is shown in Figure 4. Massive open pores exist on the surface of the pure EP residue. DOPO/EP expressed the same broken pore channels that allow the pyrolysis products to easily pass through. The introduction of AHP made the carbon layer compact with some closed-pores which was attributed to the good charring effect reflected by the above quantitative assessment of the three main flame-retardant modes. For HPDAl/EP, the compactness of the char increased with increased addition, and 3%HPDAl/EP showed an especially continuous large-area and dense carbon layer. This further suggests that the reason for the insignificant quenching-out effect of 3%HPDAl/EP in the vertical combustion test was that the enhanced carbon layer hindered the ejection of internal gases. In contrast, 2% HPDAl/EP still presented some closed and open pores. Furthermore, small particles on the char residue were observed in its local magnification photos, which might be degradation products from aluminum hypophosphite. These small particles accumulated on the surface facilitated the formation of more char residues [34]. The carbon layer, which was moderately strong and can be broken by volatile pyrolysis gases, provided a suitable environment for exerting the combined effect of gas-phase and condensed-phase actions.

### 2.6. Condensed Phase Flame Retardant Mechanism

**Figure 5:**
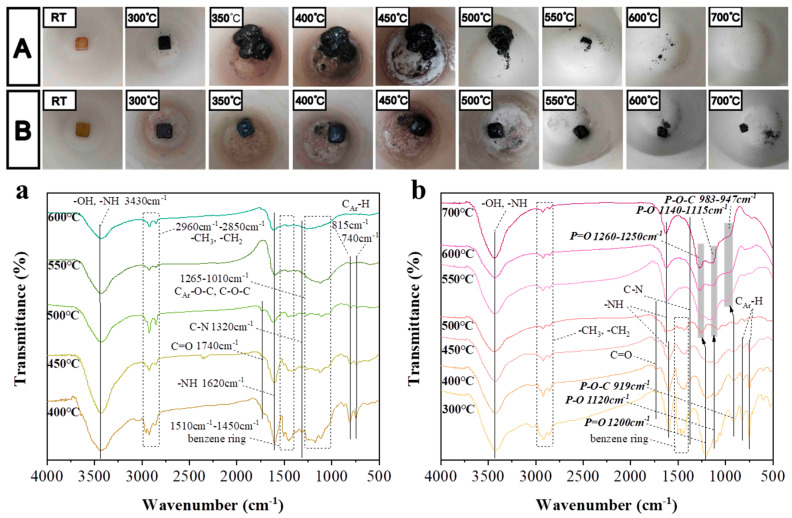
Digital photographs and residual char FTIR spectra of EP (**A**,**a**) and 2%HPDAl/EP (**B**,**b**) after being maintained at different temperatures for 15 min in a muffle furnace.

In order to observe the char formation of the EP composites during thermal degradation, pure EP and 2%HPDAl/EP were kept in a muffle furnace at various temperatures for 15 min, digital photos of which are shown in Figure 5A,B, respectively. It can be seen that the pure EP quickly expanded to the maximum at 400 °C, and the expansion of the char layer resembled the explosion of popcorn, the volume of which increased by five-fold and was beyond recognition. After 450 °C, the char residues began to decrease, and were no longer visible in the crucible at 700 °C. In contrast, 2%HPDAl/EP began to expand at 300 °C, which was earlier than the pure EP, and then grew to a maximum at 450 °C. It might be that HPDAl promoted the early decomposition of the matrix into carbon, while its release of pyrolysis gases drove the expansion of the carbon layer. The expansion process was relatively slow compared to the fast decomposition of pure EP which produced plenty of flammable volatiles within a short time and resulted in the irregular expansion of residues. Interestingly, the shape of the 2%HPDAl/EP sample after expansion did not change much and was close to inflating hexahedron, whose edges were fixed but the surfaces protruded outward. As the temperature rose to 700 °C, a considerable amount of residues was still retained. It was during the decomposition process that massive cross-linking reactions might occur from the flame retardant itself or between the flame retardant and matrix, which promoted the formation of a hard and high-temperature stable carbon layer, preventing the further degradation of the substrate and then limiting the shape of the expanded carbon layer.

Meanwhile, the FTIR tests of EP and 2%HPDAl/EP residues maintained in the muffle furnace at different temperatures were performed, and the curves are shown in Figure 5a,b. Some structural information of the char residues was revealed: -OH or -NH (3430 cm^−1^ and 1620 cm^−1^), -CH_3_ (2960 cm^−1^), -CH_2_ (2850 cm^−1^), C-O-C (1265-1010 cm^−1^), C=O (1740 cm^−1^), C-N (1320 cm^−1^), and benzene ring (1510-1450 cm^−1^, 815 cm^−1^, and 740 cm^−1^) [35]. The big difference was that P=O (1200 cm^−1^), P-O (1120 cm^−1^), and P-O-C (919 cm^−1^) appeared in 2%HPDAl/EP, and there were still intense absorption peaks up to 700 °C. This indicates that HPDAl participated in the char-forming reaction of the matrix and formed abundant cross-linking structures containing P-O-C bonds, thus strengthening the char residues. This can be attributed to the fact that HPDAl decomposed into a non-flammable viscous phosphoric acid liquid film covering the resin surface upon heating, and the glassy polyphosphoric acid produced by further dehydration polymerization of phosphoric acid was able to catalyze the dehydration of -OH-containing compounds to form char layers [36]. In addition, Al^3+^ reacted with O=P-O- to generate aluminum pyrophosphate, aluminum polyphosphate, aluminum metaphosphate, etc., further promoting the dehydration of polymer chains to form a high-quality char layer in the condensed phase.

Additionally, the element contents of the residues after being sufficiently mixed and grinded during the cone calorimeter tests were detected via X-ray photoelectron spectroscopy (XPS). Element contents and the reserved and released phosphorus (P) contents were calculated and shown in Table 5 and Table 6. XPS results indicated that the carbon residues of HPADl/EP contained elements of C, N, O, P, and Al. In Table 6, we clearly see that, for AHP/EP, most of P (77.9%) was reserved in the solid phase, improving the char formation ability, which explains why AHP/EP has a positive charring effect on other samples. However, most P in DOPO/EP was released into the gas phase, thus mainly providing a flame-inhibition effect in the gas phase. The reserved P ratio in the total P content of the physically mixing system, AHP/DOPO/EP, lay between AHP/EP and DOPO/EP, while close to the former. More than half of the total P (73.8%) was still retained in the condensed phase. This might be due to the interaction between AHP and DOPO, making it difficult for the physical combination system to exert free radical quenching effects [17]. In contrast, 2%HPDAl/EP obtained a relatively balanced distribution of phosphorus elements, reserving 53.5% P in the residues and releasing 46.5% P, which inhibited the combustion reaction in the gas phase, whilst ensuring the strength of the carbon layer in condensed phase. This coordinated the gas-phase and condensed-phase flame retardant effect, which then was conducive to enhancing the flame retardant efficiency of HPDAl in EP. As expected, the reserved P ratio in the condensed phase increased with the increasing addition amount of HPDAl and highly P-rich char residues always yielded a high viscosity/strength due to the formation of more P-containing cross-linked structures. This explained why the 3%HPDAl/EP residue exhibited a lower expansion ratio and then an inferior blown-out effect.

### 2.7. Gas Phase Flame Retardant Mechanism

**Figure 6:**
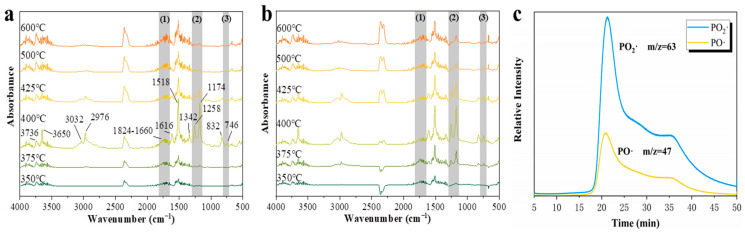
FTIR spectra of volatile pyrolysis products of EP (**a**) and 2%HPDAl/EP (**b**) at different temperatures, TG-FTIR-GC/MS curves of PO· and PO_2_· from HPDAl (**c**).

In order to illustrate the effect of flame retardants in the gas phase, TGA-FTIR tests were performed on pure EP and 2%HPDAl/EP, as shown in Figure 6a,b. For example, free NH (3735 cm^−1^) confirmed the presence of NH_3_. Olefins (3034 cm^−1^), backbone vibrations (1610 and 1512 cm^−1^), and substitutions (828 and 747 cm^−1^) absorption bands confirmed the presence of aromatic compounds. In addition, some volatiles containing -OH (3651 cm^−1^), methyl C-H (2974 cm^−1^), and C-N (1336 cm^−1^) were found. The 1820-1665 cm^−1^ absorption bands represented a substance containing C=O structure in the pyrolysis product. This part was considered a volatile fuel generated by matrix decomposition during heating, which was labeled as areas (1) and (3). Although 2%HPDAl/EP had the same position for most peaks as pure EP, it was worth noting that the absorption peak of 2%HPDAl/EP in region (1) was much weaker than that of pure EP. Furthermore, the aforementioned HPDAl itself would release carbonyl-containing substances, and then these substances would be released more reasonably after adding HPDAl, but the fact was that they decreased rather than increased. This was due to the hydroxyl groups of CH-OH in HPDAl being consumed by reacting with epoxy groups, which changed the degradation path of HPDAl. In Figure 6, 1258 and 1175 cm^−1^ corresponded to the aromatic and aliphatic C-O, respectively, which came from the degradation of EP and was labeled as region (2). Meanwhile, 1258 cm^−1^ and 747 cm^−1^ represented the P=O and o-disubstituted benzene ring structures, respectively, for 2%HPDAl/EP, both of which belonged to the characteristic absorption peaks of the phosphaphenanthrene group and its cleavage products. The absorption peaks of 2%HPDAl/EP in region (2) appeared earlier than those of pure EP, and maintained a high peak intensity after the absorption peaks of pure EP almost disappeared. This indicates that HPDAl contributed to convert the C=O groups of the pyrolysis products in epoxy thermosetting resins into a high-temperature stable P-O-C cross-linked structure. The cleavage fragments of the phosphorus heterophilic group were released into the gas phase, which provided an important material basis for the phosphaphenanthrene group to participate in and disturb the further cleavage, oxidation and combustion reactions of the volatile pyrolysis products from resin matrix. The nature of chemical moieties surrounding the phosphorus atoms in HPDAl enhanced the gas-phase and condensed-phase flame retardant effect by reducing volatile fuels.

**Figure 7:**
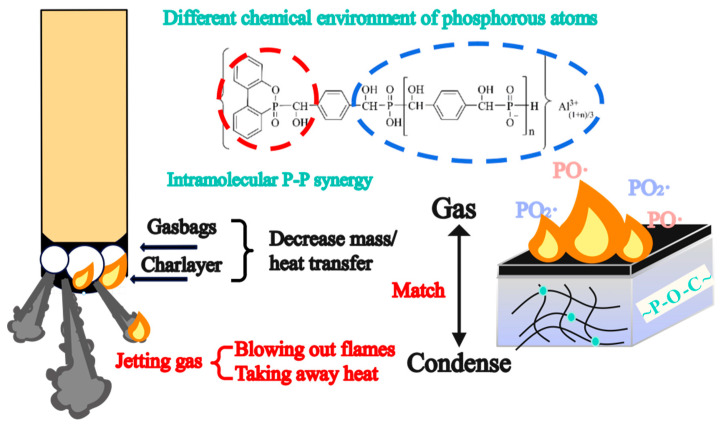
The flame retardant mechanism illustration of HPDAl in EP.

In addition, TG-GC/MS was used to track the PO· and PO_2_· released during the HPDAl pyrolysis process (50-700 °C). As seen from Figure 6c, the release of PO· and PO_2_· started at approximately 240 °C, and two peaks at 21.0 min and 35.7 min, respectively, corresponded to the first degradation peak (251 °C) and the second degradation peak (500 °C) was in the TGA curve. This clearly illustrated that HPDAl continuously released PO· and PO_2_· with high intensity during a wide temperature range of the two decomposition stages from 250 °C to 500 °C due to the pyrolysis of the phosphorus heterophilic and phosphate groups in HPDAl. The results reveal that, after HPDAl was applied to epoxy thermosetting resins, the composite material began to release PO· and PO_2_· at a relatively lower temperature and was continuously released together with the other pyrolysis products, thereby providing flame retardancy in the gas phase. Figure 7 gave the flame retardant mechanism illustration of HPDAl in EP.

### 2.8. Mechanical Properties of Flame-Retardant EP

**Figure 8:**
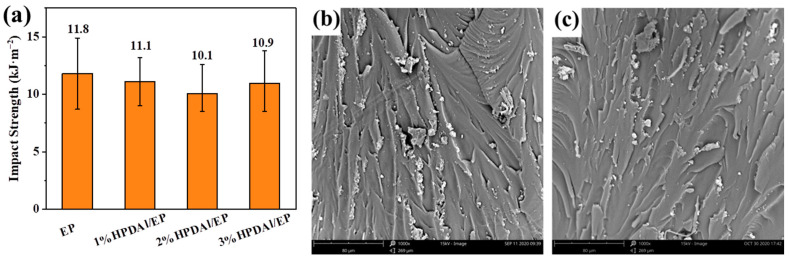
Impact strength (**a**) of epoxy composites and microtopography for the impact fractured surface of EP (**b**) and 2%HPDAlEP (**c**) composites (SEM-1000X).

The high-temperature curing epoxy thermoset materials were often required to possess certain mechanical properties. Therefore, the impact of HPDAl on the mechanical properties of epoxy composites was studied by the Charpy impact test. The results are manifested in Figure 8a. Generally, the compatibility of hypophosphite with polymers was not satisfactory, so the mechanical properties of materials would be greatly deteriorated after adding hypophosphite. However, from the results of the impact experiments, the addition of HPDAl exerted a slight influence on the mechanical properties of resin matrix. This was mainly because the HPDAl containing -OH functional groups was connected to resin chains through the curing reaction, increasing the compatibility between the flame retardant and epoxy resin, thus improving the impact resistance of composites. Furthermore, the microtopography of the impact fracture surface revealed some microstructure information, as shown in Figure 8b,c. The impact section of the pure EP exhibited a band-like texture area, accompanied by the curling of the edges, which reflected the toughness of the material. 2%HPDAl/EP had a texture similar to pure EP, which corroborated the addition of HPDAl and had no significant damage on the impact property of the matrix from the perspective of microstructure.

## 3. Methods and Materials

### 3.1. Materials

Absolute ethanol, ethyl acetate, and petroleum ether were of analytical grade, and all aqueous solutions were prepared with deionized water. Hypophosphorous acid (50% H_2_O) was supplied by Sinopharm Chemical Reagent Co., Ltd., Shanghai, China. 9,10-Dihydro-9-oxa-10-phosphaphenanthrene-10-oxide (DOPO) was provided by Shanghai Eutec Chemical, China. Aluminum chloride hexahydrate was purchased from Shanghai Aladdin Biochemical Technology Co., Ltd., Shanghai, China. 1,4-Phthalaldehyde was provided by Beijing Innochem Technology Co., Ltd., Beijing, China. 4,4′-diamino-diphenylmethane (DDM) was obtained from J&K Scientific Ltd., Beijing, China. The diglycidyl ether of bisphenol-A (DGEBA) was bought from Blue Star New Chemical Material Co., Ltd., Shanghai, China.

### 3.2. Synthesis of HPDAl

1,4-Phthalaldehyde (0.05 mol, 6.7 g) and ethanol (100 mL) were added to a three-necked flask and heated to 50 °C with mechanical stirring. After 1, 4-Phthalaldehyde was completely dissolved, hypophosphorous acid (0.05 mol, 6.6 g) was added to the flask and the temperature was increased to 90 °C for 12 h. DOPO (0.05 mol, 10.8 g) was then added to the flask and the reaction was continued for 12 h and then cooled to room temperature. AlCl_3_·6H_2_O (0.017 mol, 4.1 g) was dissolved in an appropriate amount of distilled water which was then added to the flask, and the mixture was heated at 90 °C for 8 h. Most of the solvent was removed under reduced pressure to give a yellow oily liquid, which was washed three times with an ethyl acetate/petroleum ether (1:1) mixture. Drying at 80 °C for 2 h in a vacuum oven gave a pale-yellow solid which was triturated to afford a white powder, and washed with distilled water to neutral, and then the product was vacuum dried at 80 °C for 12 h. The yield was 82%. The synthetic route of HPDAl was presented in Figure 2a. The n value representing the degree of polymerization in Figure 2a was 0.22.

### 3.3. Preparation of Flame-Retardant EP and the Control Samples

The formulations of each epoxy thermoset are listed in Table 7. HPDAl with a certain mass was added to DGEBA at room temperature, then gradually heated to 120 °C and stirred until HPDAl was completely dispersed. Thereafter, the temperature was lowered to 110 °C, and the curing agent DDM was quickly added to the mixture and stirred until it was melted. After being degassed in a vacuum oven at 120 °C for 3 min, the mixture was transferred into a preheated mold and cured at 120 °C for 2 h and then at 170 °C for 4 h. To better clarify the intramolecular P-P synergy, we prepared AHP/EP, DOPO/EP, and AHP/DOPO/EP with a phosphorus content consistent with 2% HPDAl/EP, as shown in Table 1. The control samples were prepared according to the above procedure.

### 3.4. Characterizations

#### 3.4.1. Structure Characterization

An infrared spectrometer (Nicolet iN10MX model) was used to collect the FTIR spectra (4000 cm^−1^–500 cm^−1^ wavelengths) via the potassium bromide disc. The ^13^C SSNMR and ^31^P SSNMR were detected using Bruker WB Solid State NMR Spectrometer (400 MHz).

#### 3.4.2. Flame Retardancy Performance Characterization

The limited oxygen index (LOI) was determined by Concept (UK) Limited Oxygen Index Module (Swindon, UK). The sizes of the samples were 130.0 mm × 6.5 mm × 3.2 mm based on ASTM D2863-17. The vertical burning test (UL 94) was measured using an FTT0082 instrument, requiring a sample size of 125.0 mm × 12.7 mm × 3.2 mm based on ASTM D3801-19. The forced combustion behavior was measured with an FTT0007 cone calorimeter (FTT, Derby, UK) and the external heat flux was 50 kW/m^2^. Each sample was tested at least twice and the size of the sample was 100 mm × 100 mm × 3 mm in line with ISO 5660:2015. All tests were repeated three times.

#### 3.4.3. Mechanism Research

The microscopic observations of carbon residue after cone calorimeter tests was proceeded with a Phenom™ Pro scanning electron microscope (SEM, Phenom World, Eindhoven, The Netherlands) under vacuum conditions at 15 kV. X-ray photoelectron spectroscopy (XPS) spectra were obtained using an Escalab 250Xi produced by Thermo Fisher Scientific with Al kα radiation and 2.5 kW X-ray power of and a vacuum of 2.6 × 10^−7^ Pa. The impact strength of EP composites was surveyed by a XJZ-50 digital impact test machine with a 2J pendulum based on ISO 179-1. The final results for each group of samples were taken from the average of five tests while calculating the margin of error. Samples sizes were 80 mm × 10 mm × 4 mm. Thermal decomposition was performed on a STA 8000 simultaneous thermal analyzer (Perkin Elmer, Waltham, MA, USA). The samples (8 mg) were heated from 50 °C to 700 °C at 20 °C/min in a N_2_ atmosphere. The FTIR spectra of the released pyrolysis gas were scanned by Frontier FTIR spectrometer (Perkin Elmer). Gas fragments released by HPDAl at the temperature of the maximum decomposition rate were collected by GC/MS. The specific experimental parameters are consistent with our previously published work.

## 4. Conclusions

A phosphorous-based bi-functional compound HPDAl was successfully synthesized and introduced into EP as a reactive-type FR. HPDAl consisted of two different P-groups—aluminum phosphinate (AHP) and phosphophenanthrene (DOPO)—which contained a different chemical environment of phosphorous atoms and thus exerted different FR functions with varying decomposition temperatures. With DOPO and AHP groups integrated in one compound structure, generating an intramolecular P-P synergy, HPDAl possessed a superior flame-retardant efficiency compared with DOPO or AHP separately or a physical combination of DOPO/AHP in EP. This depended on the nature change of the chemical moieties surrounding the phosphorus atom. The appropriate adding content of HPDAl brought a continuous blowing-out effect during UL 94 tests and consequently, 2 wt.% HPDAl in EP achieved a UL 94 V-0 rating with an LOI value of 32.3%. Well matching between the properties of the chars and the inner volatile pyrolysis products containing phosphorus-based radicals and nonflammable gases contributed to the more evident blowing-out effect. The investigation illustrated that the phosphorus-based flame retardant began to decompose at the early thermal degradation process of EP/HPDAl and continuously released phosphorous-based radicals during pyrolysis, exhibiting a flame inhibition effect in the gas phase. HPDAl also changed the decomposition path of the matrix, reduced the release of fuel, and suppressed the combustion from the source. The decomposition products of HPDAl in the condensed phase promoted the formation of more char residues containing the P-O-C cross-linked structures with aluminum phosphate, exerting a charring effect. This work provides an alternative access to obtain flame-retardant epoxy resins by improving the flame retardant properties without compromising the mechanical properties of the material.

## Figures and Tables

**Table 2:** Thermal behavior and charring parameters of EP composites.

Samples	N_2_ Atmosphere					Air Atmosphere				
	*T*_d,5%_ (°C)	*T*_peak_ (°C)	Char Residue (wt.%)			*T*_d,5%_ (°C)	*T*_peak_ (°C)	Char Residue (wt.%)		
			500 °C	600 °C	700 °C			500 °C	600 °C	700 °C
EP	391	405	20.5	16.3	14.9	388	398	31.7	24.6	9.3
1%HPDAl/EP	373	402	24.7	20.7	19.7	370	395	34.3	27.1	10.7
2%HPDAl/EP	367	399	25.4	21.1	20.3	361	389	34.5	27.3	11.8
3%HPDAl/EP	355	398	25.7	21.8	21.0	353	391	35.2	28.8	13.1
AHP/EP	380	401	26.4	21.2	20.1	356	396	42.3	32.1	10.8
DOPO/EP	376	400	24.8	19.6	18.5	362	390	40.7	31.4	11.1
AHP/DOPO/EP	375	398	26.6	21.5	20.4	348	395	43.9	32.8	10.5

**Table 3:** Combustion parameters collected in cone calorimetry test.

Samples	TTI (s)	PHRR (kW m^−2^)	THR (MJ m^−2^)	TSP (m^2^)	TSR (m^2^ m^−2^)	av-EHC (MJ kg^−1^)	av-COY (kg kg^−1^)	av-CO_2_Y (kg kg^−1^)	Residue (wt.%)
EP	56 ± 1	1420 ± 53	144 ± 5	52.2 ± 1.3	5770 ± 171	29.9 ± 0.3	0.13 ± 0.01	2.51 ± 0.08	7.70 ± 0.41
1%HPDAl/EP	52 ± 2	1188 ± 36	117 ± 4	41.7 ± 1.4	4722 ± 142	27.2 ± 0.1	0.12 ± 0.01	2.20 ± 0.07	12.50 ± 0.35
2%HPDAl/EP	50 ± 2	1072 ± 32	107 ± 3	40.1 ± 1.2	4536 ± 136	27.0 ± 0.1	0.13 ± 0.00	2.20 ± 0.06	14.50 ± 0.39
3%HPDAl/EP	49 ± 1	1128 ± 34	115 ± 3	41.9 ± 1.2	4740 ± 139	27.2 ± 0.1	0.15 ± 0.01	2.19 ± 0.07	14.00 ± 0.32
AHP/EP	58 ± 2	1329 ± 40	139 ± 4	46.2 ± 1.5	5097 ± 155	29.8 ± 0.2	0.17 ± 0.02	2.50 ± 0.08	15.37 ± 0.45
DOPO/EP	52 ± 2	1351 ± 41	129 ± 4	54.8 ± 2.1	6060 ± 181	27.7 ± 0.2	0.20 ± 0.02	2.29 ± 0.09	12.01 ± 0.37
AHP/DOPO/EP	56 ± 1	1223 ± 37	133 ± 4	49.1 ± 1.5	5021 ± 152	28.9 ± 0.3	0.16 ± 0.01	2.40 ± 0.06	13.89 ± 0.42

**Table 4:** Quantitative assessment of three main flame-retardant modes of action.

Samples	Flame-Inhibition Effect (%)	Charring Effect (%)	Barrier and Protective Effect (%)
1%HPDAl/EP	9.0	5.2	−2.9
2%HPDAl/EP	9.7	7.4	−1.8
3%HPDAl/EP	9.0	6.8	1.3
AHP/EP	0.3	8.3	3.4
DOPO/EP	7.4	4.7	−6.1
AHP/DOPO/EP	3.0	6.7	−0.6

**Table 5:** Elemental concentrations of residues of EP composites from XPS.

Element Contents	C (%)	N (%)	O (%)	P (%)	Al (%)
1%HPDAl/EP	88.25	1.21	9.42	0.52	0.60
2%HPDAl/EP	85.98	1.67	10.65	1.07	0.47
3%HPDAl/EP	83.27	2.71	11.67	1.98	0.44
AHP/EP	70.24	5.88	19.39	1.47	3.03
DOPO/EP	84.65	4.81	9.87	0.67	0.00
AHP/DOPO/EP	79.86	3.80	13.39	1.54	1.41

**Table 6:** Reserved and released P contents in the cone calorimeter test by XPS.

Samples	P Ratio in Residues (%)	Char Yield (%)	Initial P Ratio in Samples (%)	Reserved P Ratio in Total P (%)	Released P Ratio in Total P (%)
1%HPDAl/EP	0.52	12.50	0.14	46.4	53.6
2%HPDAl/EP	1.07	14.50	0.29	53.5	46.5
3%HPDAl/EP	1.98	14.00	0.43	64.5	35.5
AHP/EP	1.47	15.37	0.29	77.9	22.1
DOPO/EP	0.67	12.01	0.29	27.7	72.3
AHP/DOPO/EP	1.54	13.89	0.29	73.8	26.2

**Table 7:** Formulation of the epoxy thermosets.

Samples	DGEBA/DDM g/g	DDM (g)	HPDAl		AHP ^a^		DOPO (g)		P-Content (wt.%)
			(g)	(wt.%)	(g)	(wt.%)	(g)	(wt.%)	
EP	100.0/25.3	25.3	-	-	-	-	-	-	0.00
1%HPDAl/EP	100.0/25.3	25.3	1.3	1.0	-	-	-	-	0.14
2%HPDAl/EP	100.0/25.3	25.3	2.5	2.0	-	-	-	-	0.29
3%HPDAl/EP	100.0/25.3	25.3	3.9	3.0	-	-	-	-	0.43
AHP/EP	100.0/25.3	25.3	-	-	0.893	0.7	-	-	0.29
DOPO/EP	100.0/25.3	25.3	-	-	-	-	2.610	2.0	0.29
AHP/DOPO/EP	100.0/25.3	25.3	-	-	0.702	0.6	0.560	0.4	0.29

^a^: AHP refers to aluminum hypophosphite.

## Data Availability

The raw data required to reproduce these findings cannot be shared at this time as the data also forms part of an ongoing study.
